# A Trench Heterojunction Diode-Integrated 4H-SiC LDMOS with Enhanced Reverse Recovery Characteristics

**DOI:** 10.3390/mi16080909

**Published:** 2025-08-04

**Authors:** Yanjuan Liu, Fangfei Bai, Junpeng Fang

**Affiliations:** 1 The College of Electronic and Information Engineering, Shenyang Aerospace University, Shenyang 110136, China; liuyanjuan@hrbeu.edu.cn (Y.L.); baifangfei@stu.sau.edu.cn (F.B.); 2 School of Integrated Circuits, Tsinghua University, Beijing 100084, China

**Keywords:** 4H-SiC LDMOS, parasitic body diode, heterojunction diode, reverse recovery characteristics

## Abstract

In this paper, a novel 4H-SiC LDMOS structure with a trench heterojunction in the source (referred as to THD-LDMOS) is proposed and investigated for the first time, to enhance the reverse recovery performance of its parasitic diode. Compared with 4H-SiC, silicon has a smaller band energy, which results in a lower built-in potential for the junction formed by P+ polysilicon and a 4N-SiC N-drift region. A trench P+ polysilicon is introduced in the source side, forming a heterojunction with the N-drift region, and this heterojunction is unipolar and connected in parallel with the body PiN diode. When the LDMOS operates as a freewheeling diode, the trench heterojunction conducts first, preventing the parasitic PiN from turning on and thereby significantly reducing the number of carriers in the N-drift region. Consequently, THD-LDMOS exhibits superior reverse recovery characteristics. The simulation results indicate that the reverse recovery peak current and reverse recovery charge of THD-LDMOS are reduced by 55.5% and 77.6%, respectively, while the other basic electrical characteristics remains unaffected.

## 1. Introduction

Silicon carbide (SiC) Metal-Oxide-Semiconductor Field Effect Transistors (MOSFETs) are emerging as the most promising candidates for high-voltage and high-power applications [1,2,3], which are attributed to their lower on-resistance, higher breakdown voltage, faster switching speed, and superior radiation resistance [4,5]. In power electronic modules such as DC-DC and AC-DC converters, the parasitic PN junction diode inherent in MOSFETs is employed to conduct a reverse current, serving as a cost-effective solution for freewheeling diode functionality [6,7,8]. However, as a bipolar device, the parasitic PN junction diode injects a large number of carriers into the drift region during conduction. This leads to a substantial reverse recovery current and charge when the diode turns off, thereby significantly increasing the power loss in power electronic systems [9].

In recent years, researchers have devoted greater efforts to addressing the abovementioned issue. The primary approach to solving this problem involves introducing unipolar diodes that are connected in parallel with a parasitic body PiN diode. The unipolar diode must possess a lower on-state voltage, when MOSFETs operate as freewheeling diodes, they conduct first, thereby significantly reducing the number of minority carriers injected into the drift region. Consequently, both the reverse recovery current and charge are greatly reduced. To summarize, there are three main types of such structures. The first is the Schottky Barrier Diode (SBD) [10,11,12], the second is the built-in MOS-Channel Diode [13,14,15], and the third is the heterojunction diode [16,17,18].

However, the existing research has focused primarily on the 4H-SiC trench MOSFETs, with few studies examining the 4H-SiC LDMOS. Therefore, this paper explores a novel 4H-SiC LDMOS featuring a trench heterojunction diode to enhance reverse recovery performance. In recent years, the Si-SiC heterojunction LDMOS has been investigated to improve the trade-off between breakdown voltage and on-resistance [19,20,21,22,23]. Nevertheless, in these works, the MOS portion of LDMOS is fabricated from silicon, while the substrate is composed of silicon carbide—a configuration that differs from the structure examined in this paper.

In this paper, a trench heterojunction diode is introduced into the source region of an LDMOS for the first time, positioned outside the main current conduction path of the device. The heterojunction diode, composed of P+ polysilicon and an N 4H-SiC drift region, is connected in parallel with a parasitic body diode and functions to suppress the conduction of the latter. The proposed device structure exhibits improved performance in terms of reverse recovery current and charge, while maintaining unchanged basic electrical characteristics such as transfer characteristics, output characteristics, and breakdown characteristics.

## 2. Device Structure and Operation Principle

**Figure 1:**
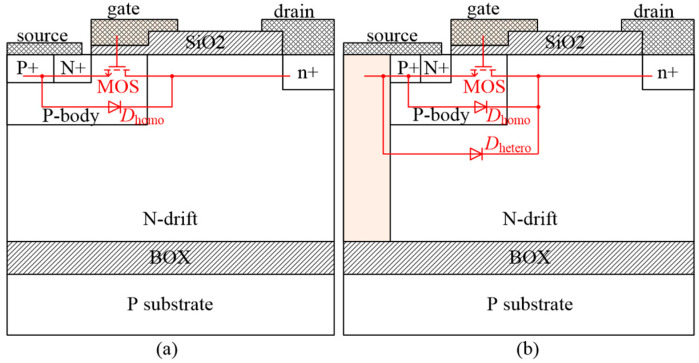
Diagram of the structures of (**a**) C-LDMOS and (**b**) THD-LDMOS.

The structures of the conventional and proposed LDMOS are presented in Figure 1a,b, abbreviated as C-LDMOS and THD-LDMOS, respectively. In addition, Figure 1 illustrates the equivalent circuits of these two structures, each containing a parasitic P-body/N-drift junction diode that is connected in parallel with the LDMOS. Compared to a C-LDMOS, the THD-LDMOS is characterized by a trench heterojunction diode (THD) in the source side, which consists of P+ polysilicon and an N-drift region. The trench heterojunction diode, a unipolar device, is connected in parallel with the body PiN diode. When the LDMOS operates as a freewheeling diode, THD conducts first due to its lower on-state voltage, while simultaneously inhibiting the conduction of the body PiN diode. Consequently, the THD-LDMOS exhibits superior reverse recovery performance.

**Figure 2:**
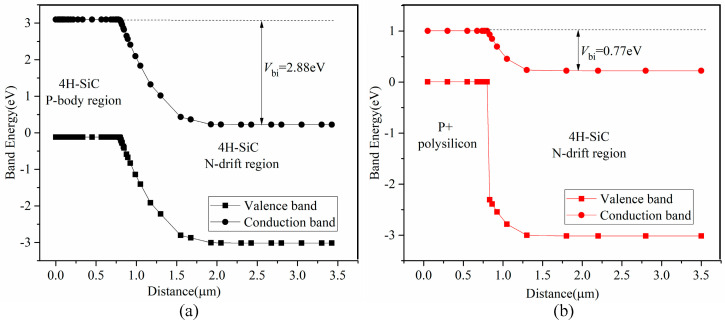
Band energy of (**a**) homojunction and (**b**) heterojunction diode at thermal equilibrium.

Figure 2 illustrates a band energy diagram of the homojunction and heterojunction diodes at thermal equilibrium. Silicon has a bandgap 1.1 of eV, approximately one-third of that of silicon carbide (3.24 eV). When a heterojunction is formed by P+ polysilicon and the 4H-SiC N-drift region, its built-in potential is approximately 0.77 V, whereas that of the 4H-SiC homojunction is about 2.88 V. Under zero bias, for both diodes, the valence band of P-type region is higher than that of the N-type region, preventing electrons in the valence band from moving from the N-type to the P-type region. Similarly, holes in the conduction band cannot migrate from the P-type to the N-type region.

**Figure 3:**
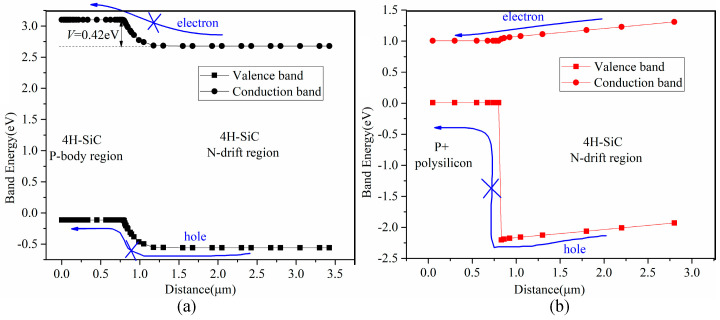
Band energy of (**a**) homojunction and (**b**) heterojunction diode at a forward-bias of 2.5 V.

A diode begins to conduct current when applied forward bias exceeds its built-in potential. Figure 3 presents the band energy diagrams of the homojunction and heterojunction diodes under a forward bias voltage of 2.5 V—a voltage greater than that of the built-in potential of the heterojunction but lower than that of homojunction. In this scenario, the valence band of the P+ polysilicon region is lower than that of the 4H-SiC N-drift region, enabling electrons in the valence band to move from the N-type to the P-type region and form an electron current. However, no hole current is generated because the conduction band of the P+ polysilicon region is higher than that of the 4H-SiC N-drift region. Thus, the heterojunction diode, similar to a Schottky Barrier Diode (SBD), operates as a unipolar device, meaning only electron carriers exist in the 4H-SiC N-drift region. This characteristic contributes to its improved reverse recovery performance. As confirmed by Figure 3a, the 4H-SiC homojunction diode remains off with no current flow when the forward bias is 2.5 V.

The C-LDMOS and THD-LDMOS share identical physical parameters, with the exception that a THD-LDMOS incorporates a trench P+ polysilicon region into its source. The P+ polysilicon trench has a depth and width of 4 μm and 2 μm, respectively. The gate oxide thickness is 30 nm, and the other parameters are listed in Table 1.

## 3. Simulation Results and Discussion

In this section, the basic electrical characteristics are investigated in detail, including the transfer characteristics, output characteristics, and body diode performance. Additionally, when the LDMOS operates as a freewheeling diode, the reverse recovery characteristics of its parasitic diode are examined in depth.

### 3.1. Basic Electrical Characteristics

#### 3.1.1. Transfer Characteristics

**Figure 4:**
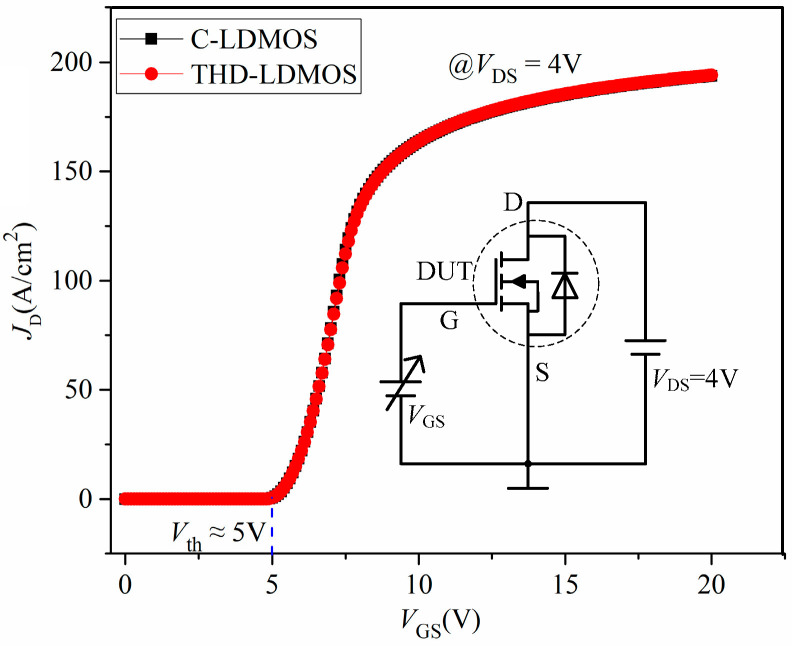
Comparison of the transfer curves.

Figure 4 presents the transfer characteristics of the two structures, illustrating the relationship between the drain current density (*J*_D_) and the gate voltage at a constant drain voltage, with the inset showing the test circuit for the transfer curves. As observed from this figure, both the C-LDMOS and THD-LDMOS exhibit a threshold voltage (*V*ₜₕ) of approximately 5 V, which can be attributed to the identical parameters of their P-body regions.

#### 3.1.2. Output Characteristics

**Figure 5:**
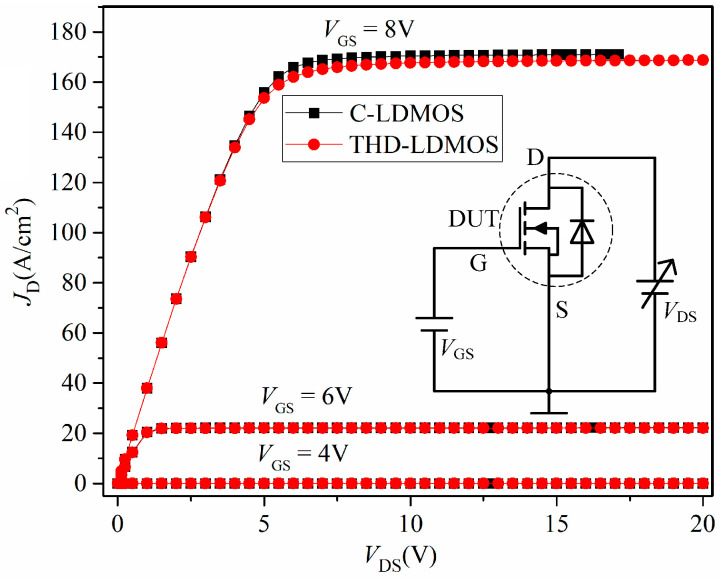
Comparison of the output curves.

Figure 5 presents a comparison of the output characteristics, illustrating the relationship between the drain current density (*J*_D_) and the drain voltage under different gate voltages, with the inset showing the test circuit for the output curves. During normal operation of the device, current flows from the drain to the source along the upper surface of the N-drift region. Since the trench P+ polysilicon is positioned outside the current flow path, the THD-LDMOS exhibits the same current-driving capability and specific on-resistance as the C-LDMOS.

#### 3.1.3. Breakdown Characteristics

**Figure 6:**
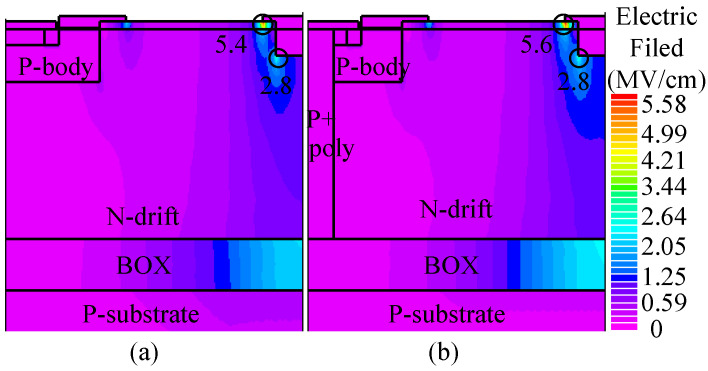
Distribution of the electric field in (**a**) C-LDMOS and (**b**) THD-LDMOS at *V*_DS_ = 600 V.

Figure 6 illustrates the electric field distribution at a drain voltage of 600 V, where the electric field strength is close to the critical breakdown field of 4H-SiC material (3 MV/cm). Both devices undergo avalanche breakdown, indicating that their breakdown voltage is approximately 600 V. As shown in Figure 6, the breakdown point is located on the drain side, while the trench heterojunction diode is positioned on the source side. Consequently, the THD-LDMOS and C-LDMOS exhibit identical voltage-withstanding capabilities.

### 3.2. Body Diode Characteristics

When the LDMOS functions as a freewheeling diode in converter applications, it is actually the parasitic body diode that acts as the freewheeling component. Therefore, it is desirable for the parasitic body diode to exhibit superior performance, such as a lower on-state voltage, lower reverse recovery current, and lower reverse recovery charge. However, the parasitic body PiN diode in the C-LDMOS is a bipolar device, which results in poor reverse recovery characteristics. Additionally, due to the higher built-in potential of the 4H-SiC diode, it has a higher on-state voltage. In contrast, the proposed structure adopts a trench heterojunction diode connected in parallel with the PiN diode, leading to improved performance.

**Figure 7:**
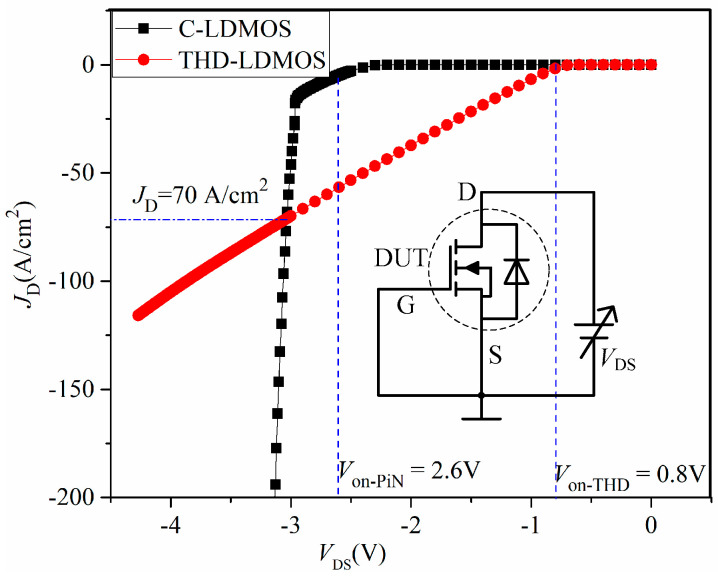
Performance of the parasitic body diode.

Figure 7 presents a comparison of the performance of the parasitic body diodes in the C-LDMOS and THD-LDMOS, illustrating the relationship between the diode current density and the forward-bias voltage. To obtain the volt–ampere characteristics of the body diode, the inset of Figure 7 shows the corresponding test circuit. The anode of the diode is connected to the source of the LDMOS, and the cathode is connected to the drain; thus, the source–drain voltage (*V*_SD_) must be negative to forward-bias the diode. As shown in Figure 7, the THD-LDMOS exhibits a lower on-state voltage when the diode current density is below 70 A/cm^2^.

**Figure 8:**
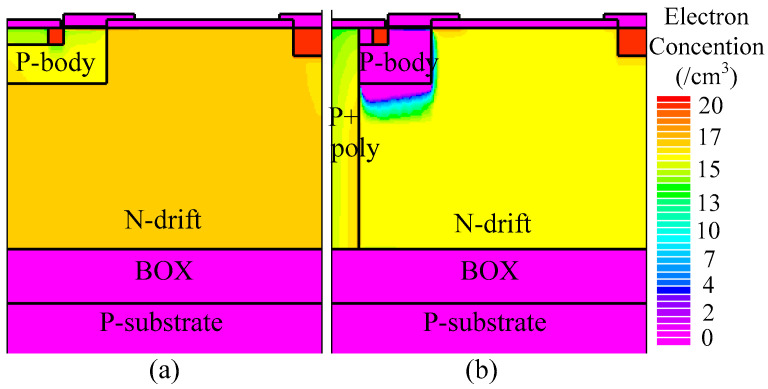
Distribution of the electron carriers in (**a**) C-LDMOS and (**b**) THD-LDMOS at *J*_D_ = 50 A/cm^2^.

**Figure 9:**
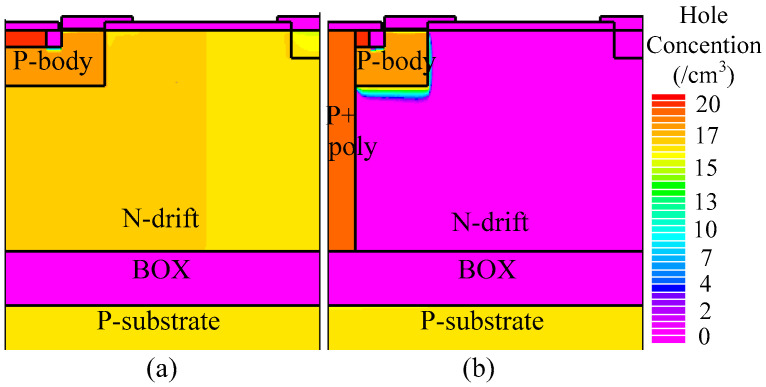
Distribution of the hole carriers in (**a**) C-LDMOS and (**b**) THD-LDMOS at *J*_D_ = 50 A/cm^2^.

**Figure 10:**
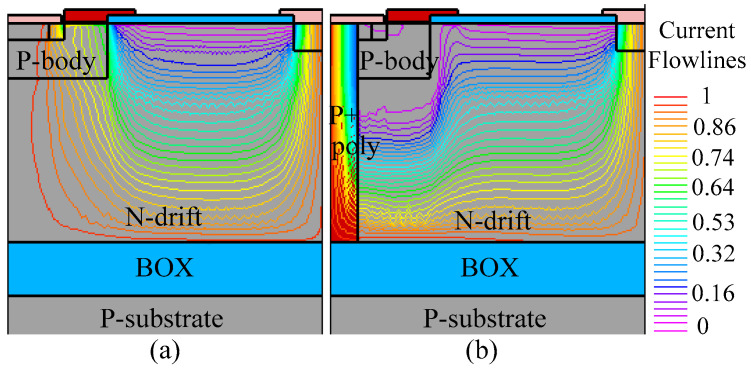
Distribution of the current flowlines in (**a**) C-LDMOS and (**b**) THD-LDMOS at *J*_D_ = 50 A/cm^2^.

For instance, when the drain current density (*J*_D_) is 50 A/cm^2^, the on-state voltage of the THD-LDMOS is approximately 2.4 V, representing a 20% reduction compared to the C-LDMOS. The primary reason for this phenomenon is illustrated in Figure 8, Figure 9 and Figure 10. The distributions of electrons (Figure 8) and holes (Figure 9) demonstrate that the trench heterojunction diode suppresses the conduction of the body PiN diode. Furthermore, the distribution of current flowlines (Figure 10) further confirms the aforementioned conclusion, as these flowlines are confined exclusively to the trench heterojunction diode.

As the forward current increases, the carrier concentration in the N-drift region of the C-LDMOS also rises, since its body diode is a bipolar device. In contrast, the carrier concentration in the THD-LDMOS remains approximately constant because its heterojunction diode operates as a unipolar device. An increase in the carrier concentration of the N-drift region leads to a reduction in its parasitic equivalent resistance. As a result, the forward on-state voltage of the proposed device (THD-LDMOS) becomes larger than that of the conventional device (C-LDMOS) under higher current conditions.

### 3.3. Reverse Recovery Characteristics

**Figure 11:**
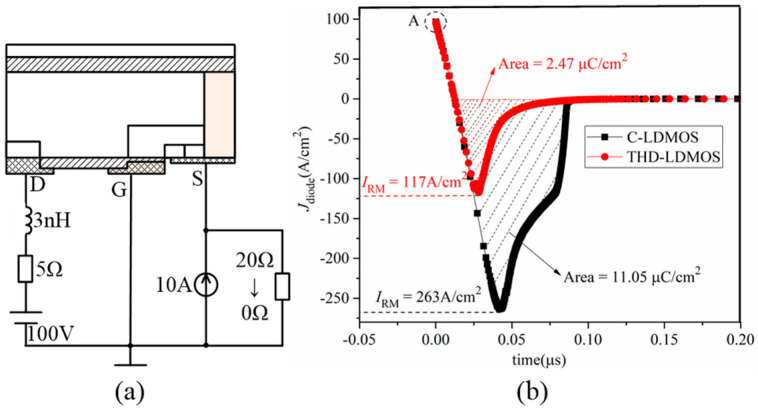
(**a**) Test circuit and (**b**) comparison of the reverse recovery characteristics at *J*_D_ = 100 A/cm^2^.

The reverse recovery performance of the parasitic diode exerts a significant influence on electronic systems utilizing an LDMOS. The test circuit is presented in Figure 11a, where a resistor and a current source are connected in parallel to control the anode voltage of the diode (the source voltage of the LDMOS). In a static state, the resistor value is 20 Ω, resulting in an anode voltage of 200 V for the parasitic diode—a value higher than the cathode voltage (or drain voltage) of 100 V—thus placing the parasitic diode in an on-state. Subsequently, the resistor value is reduced from 20 Ω to 0 Ω, causing the anode voltage of the parasitic diode to drop from 200 V to 0 V, which simulates the reverse recovery process of the diode.

The reverse recovery characteristics of the C-LDMOS and THD-LDMOS are presented in Figure 11b. As shown in this figure, the THD-LDMOS exhibits a lower reverse recovery peak current (*I*_RM_) of approximately 117 A/cm^2^, representing a 55.5% reduction compared to the C-LDMOS, which has a value of about 263 A/cm^2^. Additionally, the reverse recovery charge (*Q*_RR_) can be derived by integrating the current over time. For the THD-LDMOS and C-LDMOS, the *Q*_RR_ values are approximately 2.47 μC/cm^2^ and 11.05 μC/cm^2^, respectively, corresponding to a 77.7% reduction in the THD-LDMOS.

**Figure 12:**
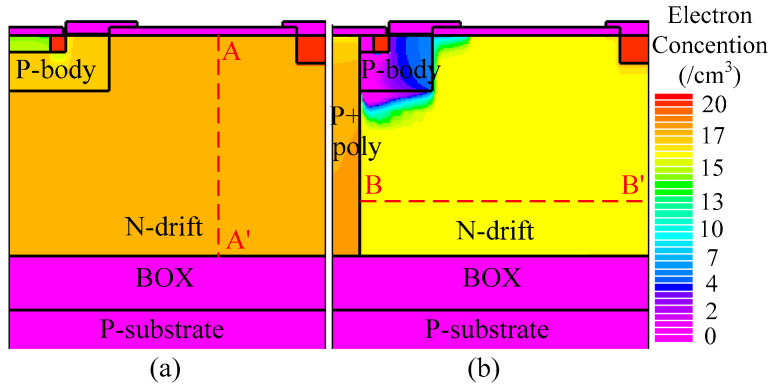
Distribution of the electrons in (**a**) C-LDMOS and (**b**) THD-LDMOS at *J*_D_ = 100 A/cm^2^.

**Figure 13:**
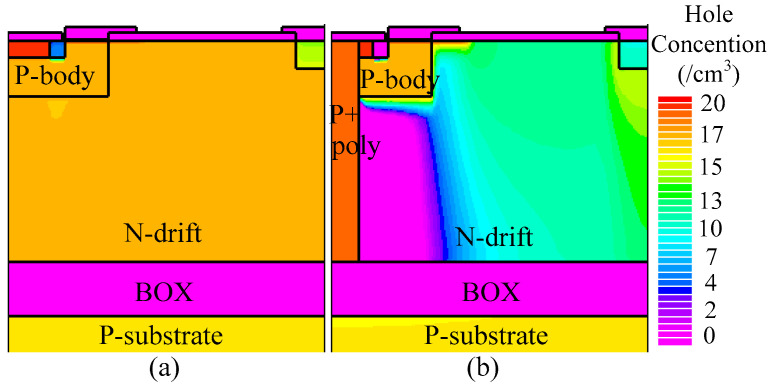
Distribution of the holes in (**a**) C-LDMOS and (**b**) THD-LDMOS at *J*_D_ = 100 A/cm^2^.

**Figure 14:**
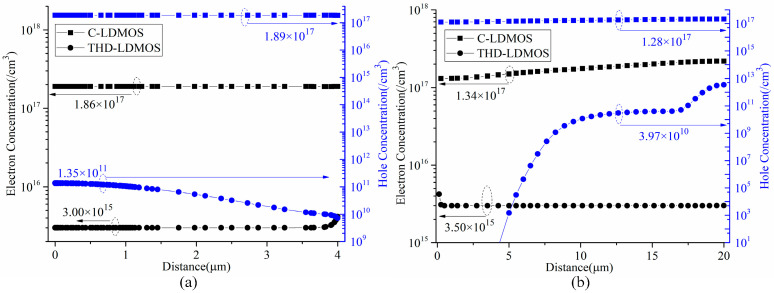
Carrier’s concentration at the cutline of (**a**) A-A’ and (**b**) B-B’ as shown in Figure 12.

Figure 12 and Figure 13 illustrate the electron and hole concentrations in the C-LDMOS and THD-LDMOS, respectively. As shown in Figure 12, at the same current density, the THD-LDMOS exhibits a lower electron concentration in the N-drift region, on the order of 10^15^ cm^−3^ (shown in Figure 14). This is 100 times smaller than the electron concentration in the C-LDMOS, which is on the order of 10^17^ cm^−3^ (shown in Figure 14). In contrast, Figure 13 reveals that the C-LDMOS contains a large number of hole carriers in the N-drift region, generated by the conduction of its parasitic PiN diode. As observed in Figure 7, although the on-state voltage of the parasitic diode in the THD-LDMOS is slightly higher than that in the C-LDMOS, its reverse recovery performance is significantly improved. This is because the trench heterojunction diode integrated into the THD-LDMOS is a unipolar device, endowing the THD-LDMOS with superior reverse recovery characteristics.

## 4. Conclusions

To enhance the reverse recovery performance of the conventional 4H-SiC LDMOS, this paper proposes a novel LDMOS device, incorporating a unipolar trench heterojunction formed by P+ polysilicon and a 4H-SiC N-drift region. For the first time, the reverse recovery characteristics of a Si/SiC LDMOS are investigated in detail. To avoid the degradation of basic electrical characteristics, the trench Si/SiC heterojunction is introduced in the source region, positioned outside the main current flow path. In summary, the proposed THD-LDMOS exhibits improved reverse recovery performance, and a lower on-state voltage for the diode compared to the C-LDMOS. Simulation results indicate that at a current density of 100 A/cm^2^, the THD-LDMOS achieves a reverse recovery peak current of 117 A/cm^2^ and a reverse recovery charge of 2.47 μC/cm^2^, representing reductions of 55.5% and 77.7%, respectively, relative to the C-LDMOS.

## Figures and Tables

**Table 1:** Key parameters of C-LDMOS and THD-LDMOS.

Symbol	Quantity	Unit
Length of channel region	3	μm
Doping of P-body region	4 × 10^17^	cm^−3^
Thickness of P-body region	1	μm
Doping of N-drift region	3 × 10^15^	cm^−3^
Width of cell	22	μm
Thickness of N-drift region	4	μm
Thickness of BOX	1	μm
Length of gate field plate	2	μm
Length of drain field plate	1	μm

## Data Availability

The original contributions presented in this study are included in the article. Further inquiries can be directed to the corresponding author.
